# Supplementary material for: Multiday cycles of heart rate are associated with seizure likelihood: An observational cohort study
Source: eBioMedicine. 2021 Oct 11;72:103619. doi: 10.1016/j.ebiom.2021.103619 (PMC8517288; doi:10.1016/j.ebiom.2021.103619)
Supplement: Supplementary file 1 [file mmc1.pdf]

Supplement

Supplementary Table 1. AEDs and seizure types of eligible participants in Tracking Seizure Cycles cohort

Participant	Anti-Epileptic Medication	Reported Types of Seizures
**P1**	Lacosamide, Brivaracetam, Zonisamide, Losartan	Aware: 23<br>Impaired awareness: 15<br>Impaired awareness; Motor: 8<br>Motor: 4<br>Motor; Aware: 17
**P2**	Carbamazepine, Clobazam	
**P3**	Levetiracetam, Topiramate, Lamotrigine	Aware: 2
**P4**	Carbamazepine, Lamotrigine, Levetiracetam, Perampanel, Zonisamide	Aware: 17
**P5**	Levetiracetam, Lamotrigine	Aware: 103
**P6**	None	Aware: 35
**P7**	Levetiracetam, Lacosamide	
**P8**	Sodium Valproate	Motor; Aware: 1
**P9**	Lacosamide, Zonisamide, Clobazam	
**P10**	Brivaracetam, Levetiracetam	Aware: 1<br>Aware; Motor: 1<br>Impaired awareness: 12<br>Motor; Impaired awareness: 16<br>Motor: 3<br>Non-Motor; Impaired awareness: 1
**P11**	Topiramate, Lamotrigine, Carbamazepine	Aware: 2<br>Impaired awareness: 18<br>Motor; Impaired awareness: 6
**P12**	Lacosamide, Zonisamide, Clobazam	
**P13**	Sodium Valproate, Lamotrigine, Perampanel	
**P14**	None	Impaired awareness: 1<br>Impaired awareness; Motor: 4<br>Motor; Aware: 1
**P15**	Sodium Valproate, Lamotrigine, Perampanel	Aware: 8<br>Motor; Aware: 1<br>Non-Motor; Aware: 3
**P16**	Levetiracetam, Topiramate, Lamotrigine	Aware: 1<br>Non-Motor; Aware: 12<br>Impaired awareness: 15<br>Impaired awareness; Motor: 1
**P17**	Zonisamide, Lacosamide	Aware: 7
**P18**	Brivaracetam, Phenobarbital, Clonazepam, Carbamazepine	Aware; Motor: 1<br>Impaired awareness: 2<br>Impaired awareness; Motor: 1
**P19**	Levetiracetam, Topiramate, Lacosamide	Aware: 3
**P20**	Sodium Valproate	
**P21**	Carbamazepine	Impaired awareness: 1<br>Impaired awareness; Motor: 11<br>Motor; Aware: 1<br>Non-Motor: 3<br>Non motor; Aware: 2
**P22**	Brivaracetam, Lacosamide, Perampanel, Lamotrigine	
**P23**	Zonisamide, Lamotrigine, Clonazapam, Medicinal Cannabis	Aware: 2<br>Aware; Non-Motor: 395<br>Impaired awareness: 5<br>Impaired awareness; Motor: 7<br>Impaired awareness; Non-Motor: 2
**P24**	Sodium Valproate, Pregabalin, Lacosamide	Motor; Impaired awareness: 4

**P25**	Sodium Valproate, Pregabalin, Lacosamide	
**P26**	Sodium Valproate, Lamotrigine	Aware: 1<br>Motor; Aware: 3
**P27**	Levetiracetam, Topiramate, Lacosamide	
**P28**	Levetiracetam, Topiramate	Impaired awareness: 1<br>Non-Motor; Impaired awareness: 3
**P29**	Clobazam, Lacosamide, Levetiracetam, Oxcarbazepine	Aware: 147<br>Motor; Impaired awareness: 1
**P30**	Oxcarbazepine	
**P31**	Lamotrigine, Clonazepam, Oxcarbazepine, Zonisamide	Aware: 22<br>Aware; Motor: 94<br>Aware; Non- Motor: 1<br>Impaired awareness: 12<br>Impaired awareness; Motor: 149<br>Motor; Impaired awareness: 78

Supplementary Table 2. Control participants in Tracking Seizure Cycles cohort

Participant	Gender	Age	Data duration (months)	Adherence (%)	Average Heart Rate (BPM)
**C1**	M	45	5	81	81
**C2**	F	39	9.8	80	71
**C3**	M	51	5.5	97	70
**C4**	F	41	15.4	86	71
**C5**	F	24	9.3	80	74
**C6**	F	44	9.6	88	77
**C7**	M	48	7.5	97	67
**C8**	M	29	8.6	83	66
**C9**	F	27	20.1	91	72
**C10**	M	30	29	96	69
**C11**	F	29	9.3	89	79
**C12**	M	42	6	96	69
**C13**	M	27	9.2	99	61
**C14**	M	24	9.2	94	75
**C15**	M	64	5.4	81	64

Appendix 1: Heart rate data interpolation

To select the best interpolation method for missing data segments, we chose a participant (P10) who had a long period without missing data, found their true heart rate cycles, and then arbitrarily removed up to 20% of the raw data (Supplementary Figure 1a). Following data removal, three different interpolation methods were applied to missing segments (Supplementary Figure 1b), cycles were detected using the Morlet wavelet and resolved cycles across interpolation methods were compared to the true cycles.

Three interpolation methods were applied to the missing data segments: ‘average time of day’ method, ‘straight line’ method and ‘copy’ method. The ‘average time of day’ method interpolated each data point with the time-matched average heart rate (e.g., a missing data point at 9AM was replaced with the average heart rate at 9AM across the whole dataset). The ‘straight line’ method interpolated each data point with the average heart rate found across the whole dataset. The ‘copy’ method interpolated each data point with the time-matched most recent heart rate values (e.g., a missing data segment from 9AM-3PM on January 1, 2020 was replaced with the data from 9AM-3PM on December 31, 2019).

To compare the interpolation methods, the Morlet wavelet was used to determine significant cycles in the data. Cycles were compared among interpolation methods to the true cycles (original data peaks detected in the Morlet wavelet spectrum). After 1000 runs of arbitrary data removal, 92.1%, 93.5% and 95.9% of true cycles were resolved and 0.40, 0.49 and 0.86 extra peaks were detected using the 'average time of day', 'straight line' and 'copy' methods, respectively. The 'straight line' method was chosen for its simplicity and overall performance while minimising erroneous cycle detections.

To further investigate the effect of the 'straight line' interpolation on cycle detection, we performed another simulation on three datasets from two other participants (P1 and P7), where a four-month period existed with no missing data. To simulate the worst-case scenario, we systematically removed between 1 and 30 segments (increasing by one segment each simulation), where deleted segment lengths were arbitrarily chosen such that the total removed time was always 20% of the total recording time. The results of simulation are shown in Supplementary Table 3. On average over 75% of actual peaks (cycles) were detected despite interpolation. Two example simulations are shown in Supplementary Figure 2, where one large segment (20% of the data length) and 15 shorter segments (arbitrary lengths totalling 20% of the data length) were removed. Both simulations retrieved cycles (Supplementary Figs 2d and 2f) that were similar to the true wavelet (Supplementary Figure 2b). The simulation of one removed segment detected cycles that were similar to the true cycles, despite a large missing chunk. However, the simulation of 15 removed chucks missed a true cycle at 72h, which was much weaker than the other peaks in the original wavelet. Overall, stronger peaks in the initial wavelet were typically detected in all simulations (regardless of length and number of missing segments), and weaker peaks were more vulnerable to going undetected.

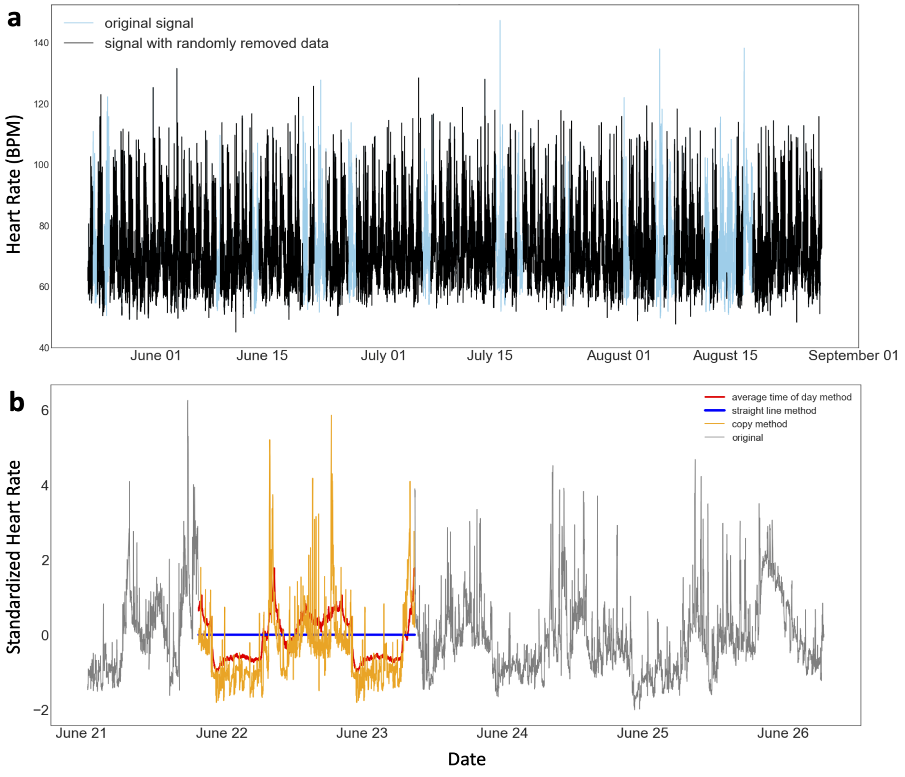

Supplementary Figure 1. Interpolated data simulation results for participant P10. a: Example simulation where up to 20% of the data was arbitrarily removed. **b:** Example missing segments with three interpolation methods: average time of day method (red), straight line method (blue) and copy method (orange).

Supplementary Table 3. Results of interpolated data simulation experiment on three segments without missing data. Two participants (P1 and P7) were used who had data segments of at least four months with no missing segments (note that P4 had two, non-overlapping four-month segments with no missing data)

	**Segment duration (days)**	**Total time removed in simulation (hours)**	**Average true peak resolution (SD)**
**P1**	273	1308	75% (17%)
**P7**	208	998	84% (12%)
**P7**	146	698	79% (19%)

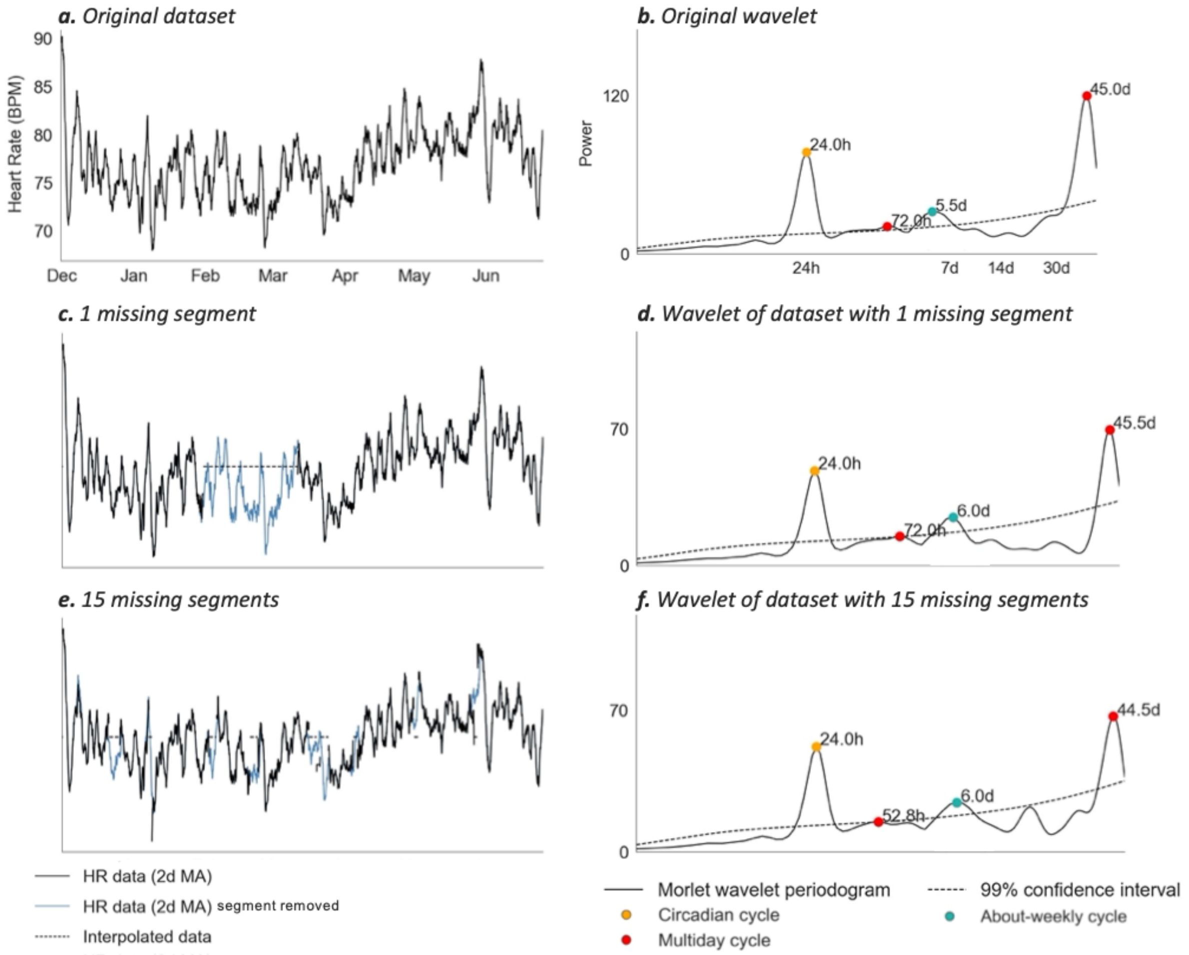

Supplementary Figure 2. Changes in cycles detected in P7's wavelet spectrum after data removed and interpolated. a: Heart rate (HR) data segment (208 days) with no missing data **b:** Wavelet spectrum of original dataset. **c,d:** HR data after segments (**c:**1 segment of 998 hours, **d:**15 segments totalling 998 hours) were arbitrarily removed. Removed segments are shown in blue and interpolation values are given by the dotted line. **e,f:** Wavelets after data was removed and interpolated with the average straight line. Note the similarly in cycles (wavelet peaks) retrieved after large segments of data are interpolated.

Appendix 2: Detection of multiday heart rate cycles

Prior to wavelet analysis the heart rate signal (post-interpolation) was z-standardised (by subtracting the mean and dividing by the standard deviation). A continuous Morlet wavelet transform with increased spacing was used on standardised data segments to compute the power at different scales (cycle periods).[1] The scales were every 1.2 hours between 2.4 and 31.2 hours, every 2.4 hours between 33.6 and 48 hours, every 4.8 hours between 52.8 and 4 days and every 12 hours between 5 days and up to a maximum period of one quarter of the recording duration. The data were then represented as a global wavelet spectrum of power (the product of the average of the square of absolute value of complex wavelet coefficients and the variance of the time series) for each scale (cycle). Peaks in the wavelet spectrum were found by comparing neighbouring values. Peaks above the global significance (99% confidence) level were determined to be significant heart rate cycle periods using a time-averaged significance test [1].

Supplementary Figure 3 shows heart rate recordings and significant detected heart rate cycles (wavelet spectrum) for all eligible participants. Supplementary Figure 4 shows the wavelet spectrums of each participant overlaid with the Fast Fourier Transform (FFT) spectrum for each participant. Supplementary Figure 5 shows an example of heart rate cycles for two people in the control cohort (C8 and C9).

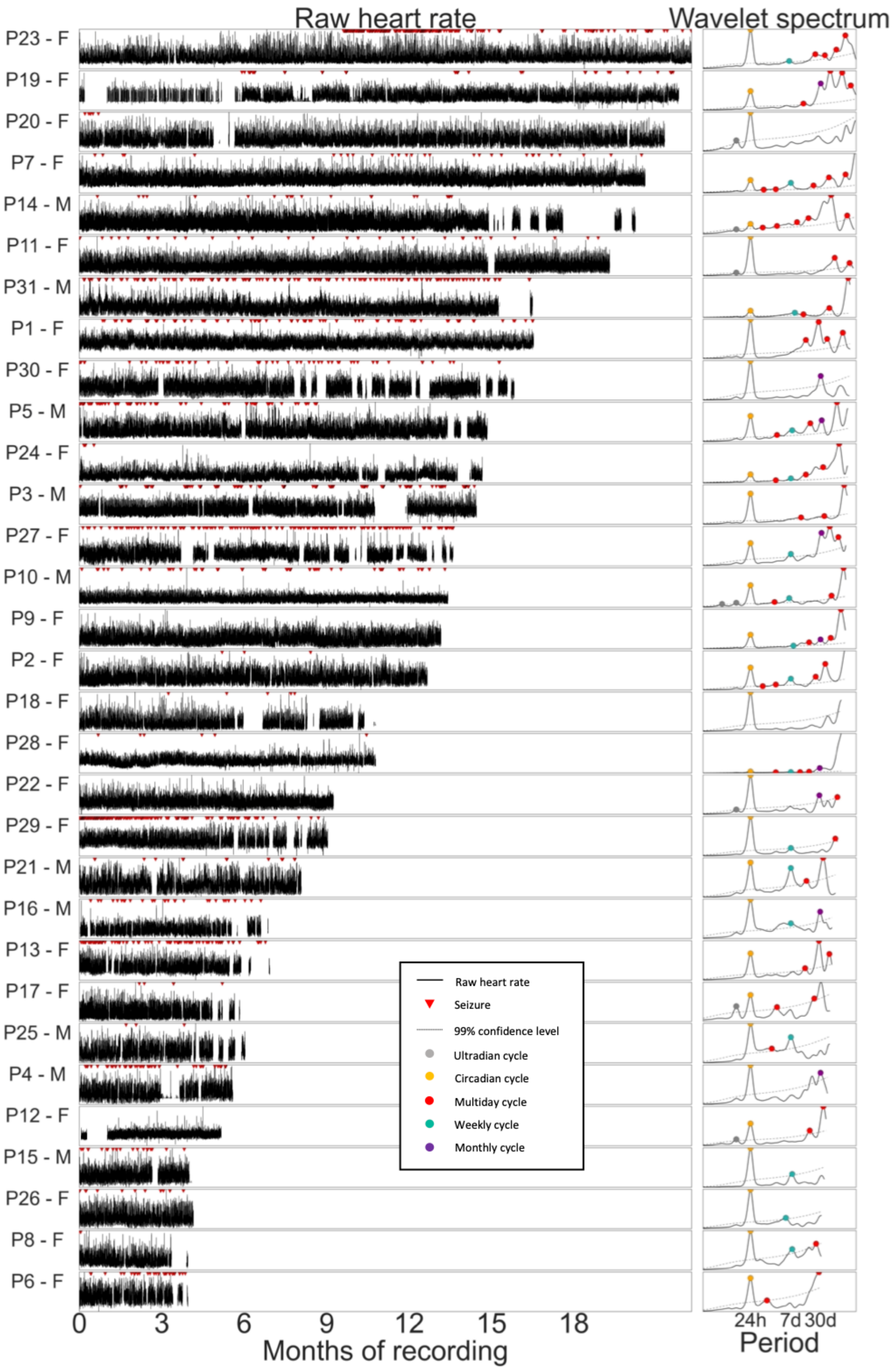

Supplementary Figure 3. Individual raw heart rate recordings and wavelet spectra for 31 participants with epilepsy. Raw heart rate data (black) are shown on the left, with seizures shown as red triangles at the top. Wavelet spectra are shown on the right with significant cycles labelled: ultradian (grey), circadian (orange),

multiday (red), weekly (turquoise) and monthly (purple). Global confidence levels are represented on the wavelet spectra by dashed black lines.

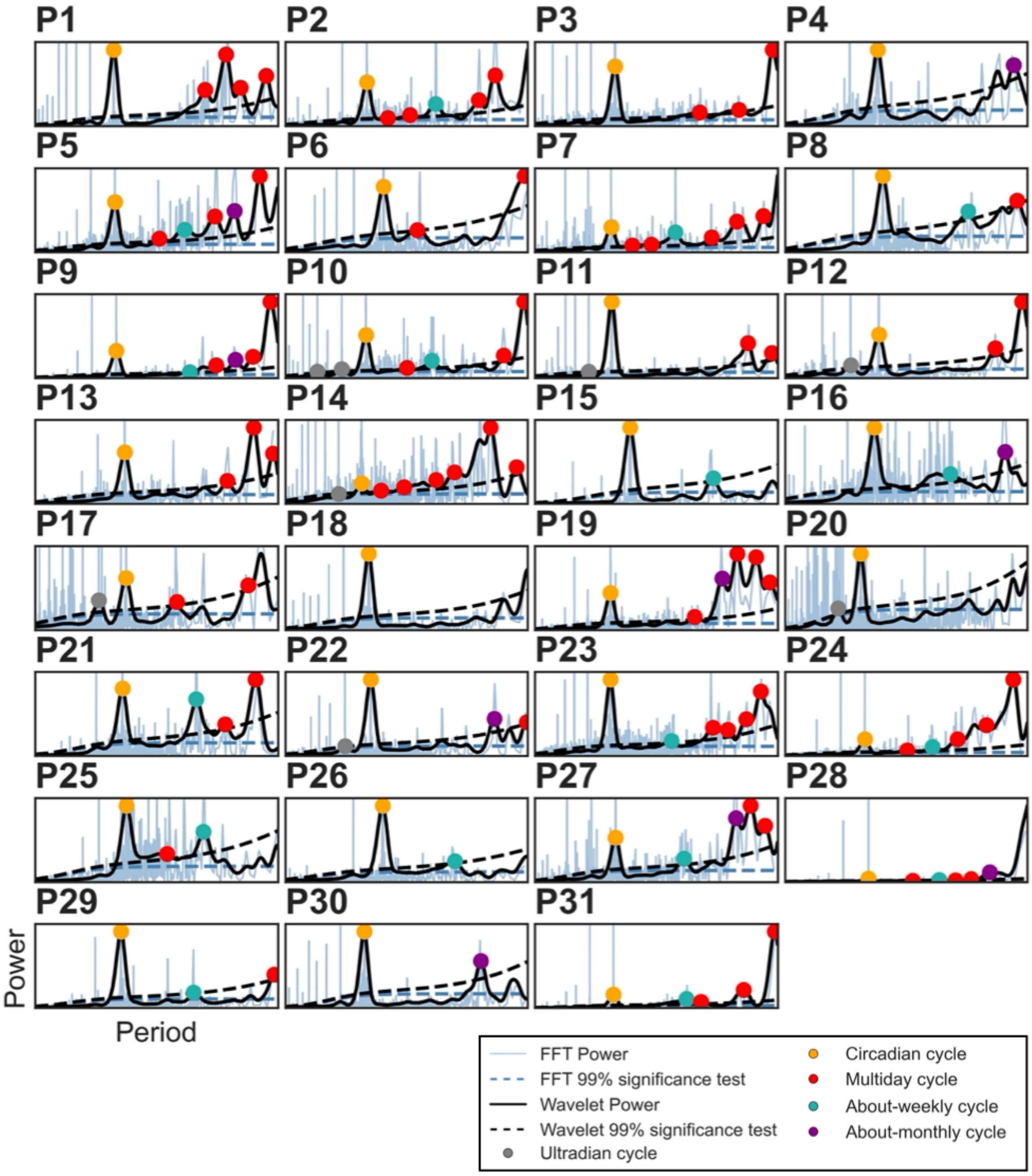

Supplementary Figure 4. Individual wavelet and Fast Fourier Transform (FFT) spectra for 31 eligible participants with epilepsy. Wavelet spectra (black solid lines) are shown with significant cycles labelled (coloured dots): ultradian (grey), circadian (orange), multiday (red), weekly (turquoise) and monthly (purple). FFT spectra are represented by the solid blue lines. Peaks in wavelet spectra correspond to peaks in FFT spectra, but wavelet spectra typically have fewer significant peaks. Global significance levels (99% confidence) are shown for both the wavelet (dashed black lines) and FFT spectra (dashed blue lines).

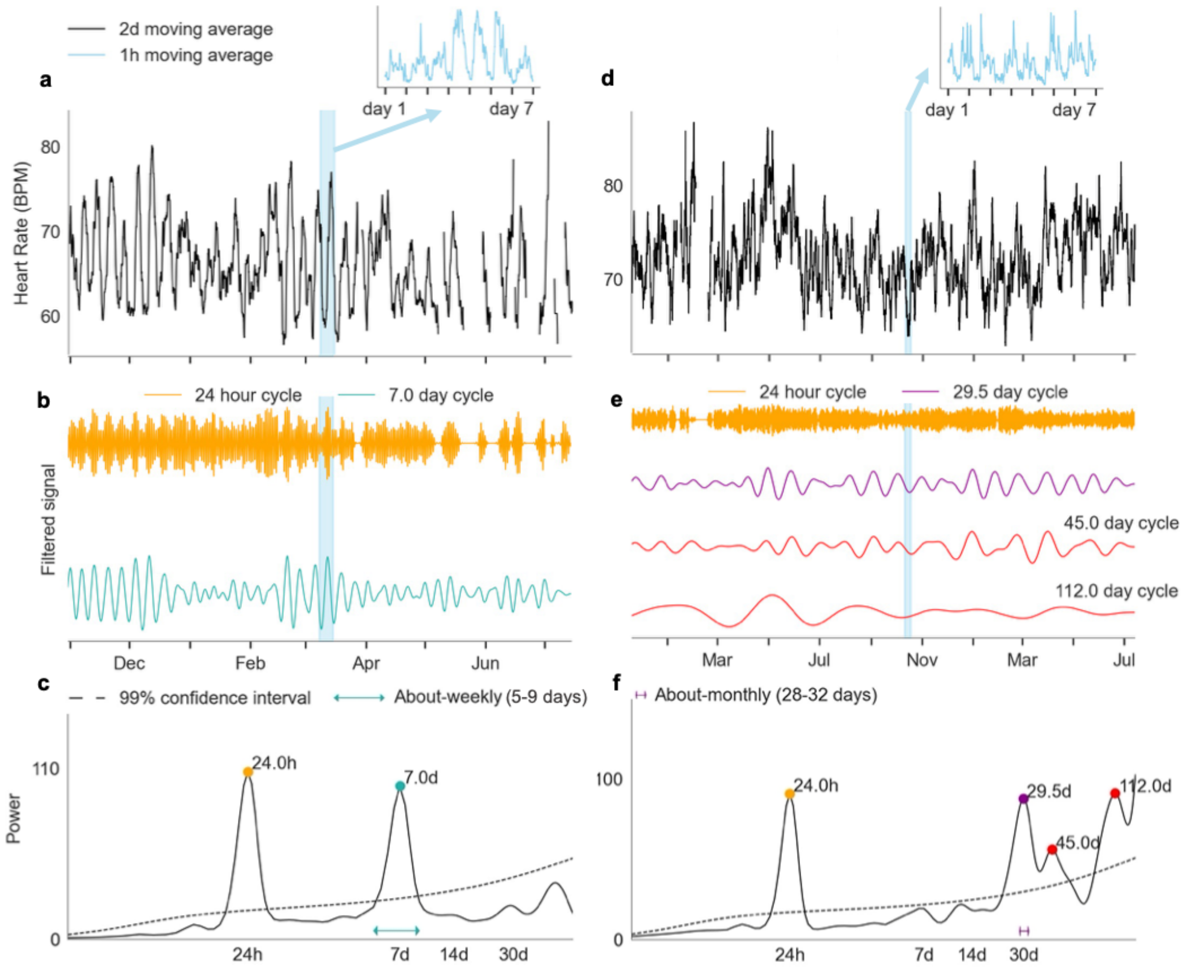

Supplementary Figure 5. Examples of multiday heart rate cycles in people without epilepsy. Data are shown for two different participants without epilepsy, C8 (a-c) and C9 (d-f). **a,d:** Heart rate (y-axis) smoothed with a 2-day moving average filter shows multiday cycles. Insets (blue) show circadian rhythms of heart rate. **b,e:** A graphical representation of the bandpass filtered heart rate signals for different cycles (corresponding respectively to spectrum peaks in panels c,f). Note that the signal amplitudes for different cycles (coloured traces) have been normalised to have the same range. **c,f:** Wavelet power spectra for different scales (x-axis). Significant cycle periods (peaks) are labelled with coloured dots.

Appendix 3: Phase locking of seizures to heart rate cycles

An omnibus (Hodges-Ajne) test[2] was used to determine whether seizures were significantly phase-locked to the heart rate cycle by testing the null hypothesis that the phase distribution was uniform. The Bonferroni correction[3] was used to account for comparisons across multiple heart rate cycles. For seizures significantly phase locked to heart rate cycles, we compared the measured SI values to the range of SI values (5th – 95th percentiles) obtained from a surrogate distribution of 100 shuffled seizure times, obtained by shuffling the inter-seizure intervals. Shuffling inter-seizure intervals randomises seizure times, while somewhat maintaining their tendency to cluster (i.e. retaining short intervals), and has been proposed as a method to determine whether the observed phase locking is meaningful compared to random phase locking that arises due to clustering[4]. Although it is important to confirm the observed effect sizes beyond what is expected by random chance, it must also be noted that shuffling inter-seizure intervals may not be a suitable randomisation if seizure timing itself is cyclic, rather than Poisson. In general, seizure times are not well described by Poisson dynamics; hence, the analysis of surrogate (shuffled) time series here should not be taken as a definitive assessment of the significance of seizure phase locking. Further work is required to develop appropriate statistical methods for assessment of seizure cycles that account for the unique dynamics of inter-seizure intervals, in particular, clustering[5], periodicity[6] and long-memory[7].

Supplementary Tables 4 and 5 show Bonferroni corrected and observed p-values for individuals' cycles (multiday and circadian, respectively), as well as the observed SI, circular mean (i.e., the angle of the mean resultant vector, indicating the direction of phase locking) and 5th – 95th percentile range for the SI of the surrogate distributions. Out of the 10 individuals with seizures significantly phase-locked to a multiday cycle, five people had SI values larger than the 95th percentile of shuffled distributions. For people with circadian or ultradian cycles, 14 out of 15 people had SI values larger than the 95th percentile of shuffled distributions.

Supplementary Table 4. Multiday heart rate cycles where seizures are significantly phase locked. Each cycle is shown with the p-value required for significance (after Bonferroni adjustment for multiple comparisons test on p = 0.05), the p-value of the seizure distribution (always less than the p-value required for significance), the SI value and the circular mean (between 0 and 2π).

Participant	Cycle (hrs)	Peak type	P-value required	P-value	SI value	SI range (5th-95th percentile) of shuffled distribution	Circular mean (radians)
**1**	648	multiday	0.01	0.001	**0.27**	0.04-0.25	1.6
	348	multiday	0.01	1.10E-10	**0.56**	0.04-0.27	2.5
**3**	2244	multiday	0.013	5.60E-05	0.11	0.09-0.46	1.2
	276	multiday	0.013	1.50E-08	**0.3**	0.04-0.27	0.35
	852	multiday	0.013	3.60E-08	0.31	0.06-0.4	0.62
**5**	180	weekly	0.0083	0.00099	0.21	0.02-0.24	2
**14**	396	multiday	0.0063	0.0043	**0.52**	0.1-0.5	4.5
**19**	2028	multiday	0.0083	0.0038	0.23	0.11-0.6	1.9
	3084	multiday	0.0083	8.60E-13	0.46	0.11-0.64	2.5
	720	monthly	0.0083	4.30E-07	0.36	0.09-0.49	3.4
	1140	multiday	0.0083	0.0018	0.21	0.06-0.46	3.5
	312	multiday	0.0083	0.00085	0.21	0.04-0.45	1.7
**21**	360	multiday	0.013	0.00038	0.66	0.3-0.84	5
**23**	552	multiday	0.0083	0.00042	0.14	0.04-0.22	0.01
	2376	multiday	0.0083	0.00018	0.13	0.05-0.31	5.7

	1524	multiday	0.0083	1.10E-05	0.12	0.05-0.33	0.52
	156	weekly	0.0083	0.008	0.07	0.02-0.14	1.3
**27**	1692	multiday	0.01	0.0083	**0.23**	0.03-0.18	1.4
**29**	1464	multiday	0.017	7.10E-08	0.21	0.04-0.26	1.8
**31**	1092	multiday	0.01	0.002	0.14	0.03-0.18	2.9
	204	weekly	0.01	4.10E-17	**0.44**	0.03-0.19	2.6
	2712	multiday	0.01	3.30E-06	0.16	0.06-0.27	4.7
	312	multiday	0.01	6.00E-06	**0.23**	0.03-0.22	2.5

Supplementary Table 5. Circadian and ultradian heart rate cycles where seizures are significantly phase locked. Each cycle is shown with the p-value required for significance (after Bonferroni adjustment for multiple comparisons test on p = 0.05), the p-value of the seizure distribution (always less than the p-value required for significance), the SI value and the circular mean (between 0 and 2π).

**Participant**	**Cycle (hrs)**	**Peak type**	**P-value required**	**P-value**	**SI value**	**SI range (5th-95th percentile) of shuffled distribution**	**Circular mean (radians)**
**1**	24	circadian	0.01	1.90E-08	**0.41**	0.03-0.21	3.4
**3**	24	circadian	0.013	8.60E-07	**0.21**	0.03-0.21	3.7
**4**	24	circadian	0.025	5.70E-06	**0.44**	0.03-0.31	3.3
**5**	24	circadian	0.0083	6.00E-17	**0.55**	0.02-0.21	2.4
**7**	24	circadian	0.0071	0.0019	**0.61**	0.05-0.35	1.4
**10**	6	ultradian	0.0071	6.30E-07	**0.47**	0.1-0.33	2.3
	12	ultradian	0.0071	8.50E-06	**0.46**	0.05-0.29	3.1
**11**	24	circadian	0.013	6.00E-05	**0.49**	0.06-0.32	2.9
**13**	24	circadian	0.013	9.30E-18	**0.44**	0.04-0.24	3.5
**16**	24	circadian	0.017	0.0084	0.4	0.07-0.47	2.9
**19**	24	circadian	0.0083	4.20E-08	**0.33**	0.03-0.3	2.6
**23**	24	circadian	0.0083	1.00E-45	**0.51**	0.03-0.13	2.8
**27**	24	circadian	0.01	4.60E-12	**0.44**	0.04-0.23	3.7
**29**	24	circadian	0.017	7.10E-08	**0.29**	0.02-0.17	3.3
**30**	24	circadian	0.025	0.0068	**0.35**	0.05-0.29	1.7
**31**	24	circadian	0.01	6.60E-15	**0.4**	0.03-0.18	1.8

Supplementary Table 6 shows the breakdown of demographic factors for individuals with seizures phase-locked to heart rate cycles. Of the participants eligible for seizure phase locking analysis (with more than 20 seizures), there were only two cases of generalised epilepsy, one focal and generalised, one non-temporal lobe epilepsy, two multi-focal, and two unknown or unclear localization. Of these, the individuals with focal and generalised epilepsy and with frontal lobe epilepsy showed seizure phase-locking to their multiday cycles. The cohort size is too small to draw any conclusions about the relationship between epilepsy syndromes and cycles; although the phenomenon was not exclusive to temporal lobe epilepsy.

Supplementary Table 6. Demographics of eligible participants with seizures phase-locked to heart rate cycles.

**Participant**	**Gender**	**Age**	**Epilepsy Type**	**Seizures**	**HR cycles with phase locked seizures**
P1	F	83	Focal (T)	105	24h, 14.5d, 27d
P3	M	55	Focal (T)	213	24h, 11.5d, 35.5d, 3.3m
P4	M	27	GGE	65	24h
P5	M	27	Focal (TO)	120	24h, 7.5d
P6	F	33	Multi-focal	37	

P7	F	30	Focal	29	24h
P10	M	69	Focal	67	6h, 12h
P11	F	55	Focal (T)	32	24h
P13	F	29	GGE (JAE)	169	24h
P14	M	70	Focal (T)	24	16.5d
P15	M	60	Focal	20	
P16	M	36	Focal (TP)	29	24h
P19	F	20	Focal (T)	103	24h, 13d, 30d, 47.5d, 3m, 4.6m
P21	M	31	Focal (T)	21	15d
P23	F	40	Focal (T)	416	24h, 6.5d, 23d, 3.5m, 2.3m
P27	F	27	Focal (T)	148	24h, 2.5m
P29	F	26	Focal (F)	251	24h, 2.2m
P30	F	29	Multi-focal	58	24h
P31	M	36	Focal and Generalized (DEE)	286	24h, 8.5d, 13d, 45.5d, 4m

Appendix 4: Phase locking to time of day and day of week

In addition to the effect size of phase-locking we considered potential biases by entrainment of seizures to time-of-day and day-of-week. The circadian modulation of seizure occurrence could not easily be differentiated between circadian cycles of heart rate versus clock time. Supplementary Figure 6 shows phase locking of self-reported seizures with respect to time of day in 14 people with more than 20 seizures and significant phase locking to their circadian heart rate cycle. 72% of seizures were reported during 8AM and 8PM.

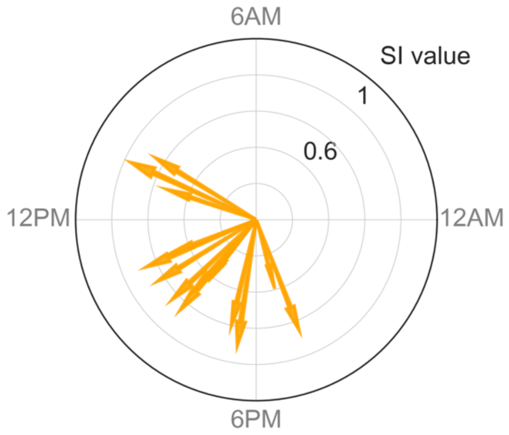

Supplementary Figure 6. Phase locking of self-reported seizures to time of day. Each arrow represents an individual (cohort of people with more than 20 seizures, N=19) with significant phase locking of their self-reported seizures to a particular time of day (N=14). The length of the arrows represents the strength of the phase locking (synchronization index, SI, value between 0 and 1) and the directions of the arrows indicate the peak times of seizure occurrence.

The tendency of seizures to occur on a particular day of the weekly heart rate cycle was also studied. Supplementary Figure 7 shows the empirical probabilities of seizures occurring on a particular day of the week for people with precise 7-day heart rate cycles (N=9). There was no evident trend for a preferred day/s on which more seizures were reported.

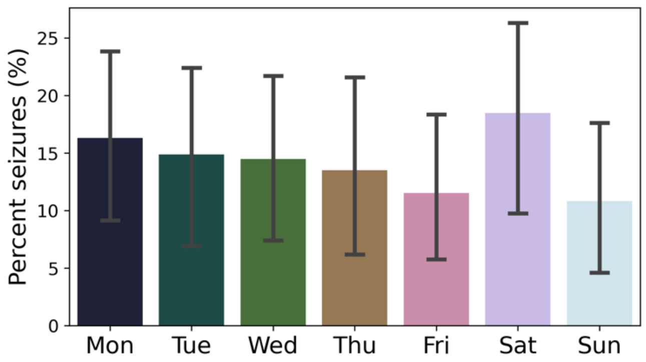

Supplementary Figure 7. Seizure probability on days of week. Empirical probabilities from seizure diaries are given for the cohort of people with precisely 7-day heart rate cycles (N = 9). Empirical probabilities shown are the mean and standard error of normalized individual seizure distributions across the population. Normalizing the individual distributions ensured people with more seizures did not bias the results.

Appendix 5: Pre- and post-ictal heart rate

Supplementary Figure 8 shows the value of pre-ictal (1-hour before reported seizure times) and post-ictal (1-hour after reported seizure times) heart rate compared to the baseline average heart rate with respect to time of day. There was no indication of consistent pre-ictal changes in heart rate across participants.

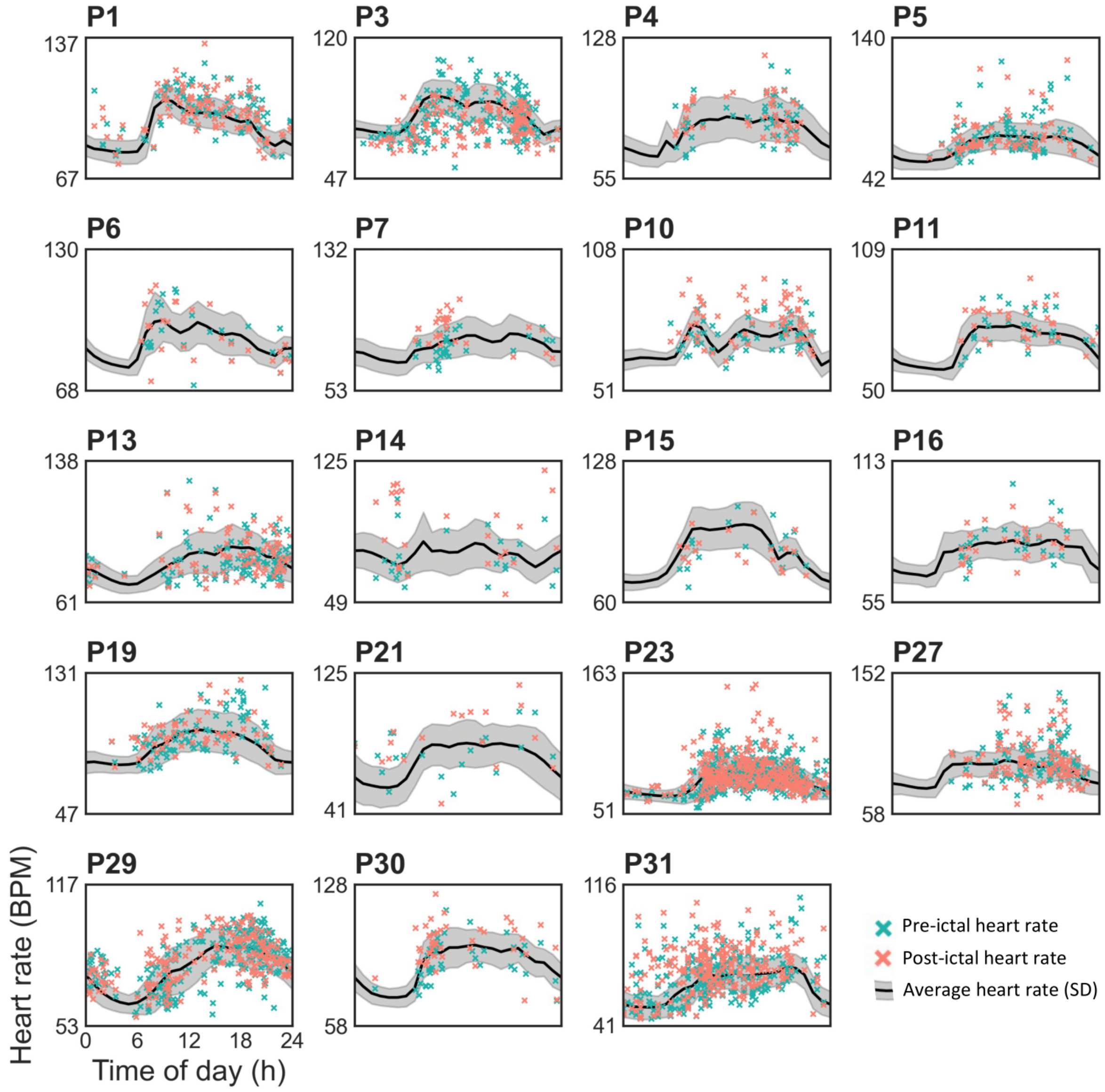

Supplementary Figure 8. Individual pre- and post-ictal heart rate analysis for 19 eligible participants with epilepsy (people with more than 20 seizures)**.** Black lines (shading) show average (standard deviation) heart rate at different times of the day across whole dataset, excluding pre- and post-ictal seizure times. Green crosses show average pre-ictal heart rate for each seizure reported. Orange crosses show average post-ictal heart rate for each seizure reported. Pre- and post-ictal are defined as 1 hour before and after a seizure was reported, respectively. Note that all x axes are the same (time of day).

Appendix 6: Reported seizure type analysis

There were six patients who reported at least 20 seizures of one seizure type (enough for seizure phase locking analysis). Of these six patients, 38 heart rate cycles were analysed and 11 were found to be comodulated with a reported seizure type. The p- and SI-values, alongside the seizure type, of each of these 11 cycles are shown in Supplementary Table 7. Of the 11 cycles, one had a lower (more significant) p-value when categorised by seizure type, and eight had higher SI-values when categorised by seizure type, compared to including all seizure types (Supplementary Tables 4-5). Importantly, all significant cycles categorised by seizure type were also significant in the analysis with all seizure types. However, the small sample size of people who had reported at least 20 seizures of one type is insufficient to draw

any conclusions about the relationships between seizure types and phase-locking to heart rate cycles.

We also investigated the relationship between seizure type and pre- and post-ictal heart rate (Supplementary Figure 9). No clear relationships were seen.

Supplementary Table 7. Heart rate cycles where seizures are significantly phase locked, grouped by reported seizure type. Each cycle is shown with the p-value required for significance (after Bonferroni adjustment), the p-value of the seizure distribution and the SI value.

Seizure type	**Participant**	**Cycle (hrs)**	**Peak type**	**P-value required**	**P-value (P-value with all seizure types)**	**SI value (SI value with all seizure types)**
Aware	1	24	circadian	0.01	0.0072 (1.9e-08)	0.42 (0.41)
		348	multiday	0.01	0.0072 (1.1e-10)	0.58 (0.56)
	5	24	circadian	0.0083	1e-08 (7.1e-08)	0.5 (0.29)
	29	24	circadian	0.017	4.7e-06 (6.0e-17)	0.35 (0.55)
Aware, Motor	31	24	circadian	0.01	1.2e-06 (6.0e-6)	0.43 (0.23)
Aware, Non-Motor	23	2376	multiday	0.0083	0.0037 (0.0002)	0.13 (0.13)
		1524	multiday	0.0083	0.0017 (1.1e-05)	0.11 (0.12)
		24	circadian	0.0083	1.5e-37 (0.008)	0.51 (0.07)
Impaired awareness, Motor	31	204	weekly	0.01	2.7e-14 (4.1e-17)	0.59 (0.44)
		24	circadian	0.01	1.1e-07 (6.6e-15)	0.44 (0.4)
		312	multiday	0.01	2.5e-05 (6.0e-06)	0.34 (0.23)

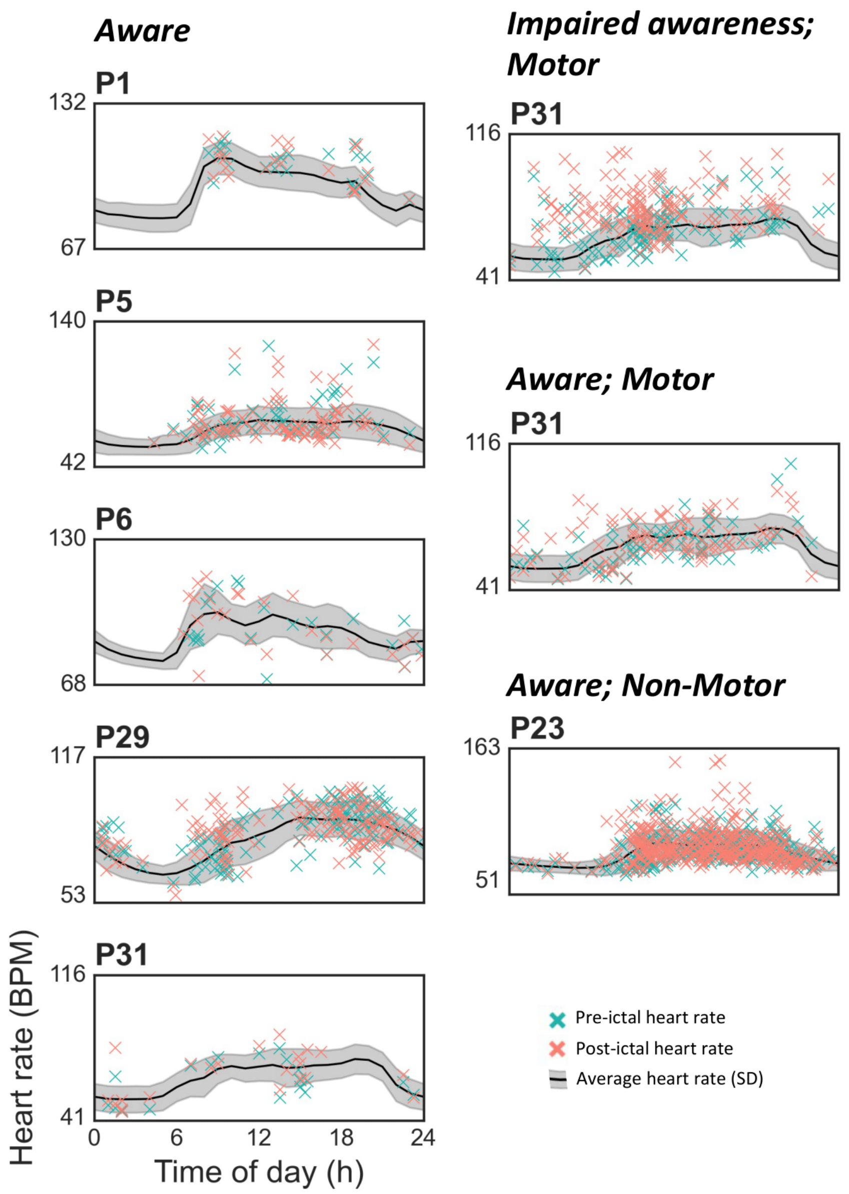

Supplementary Figure 9. Pre- and post-ictal heart rate analysis with respect to seizure type (people who reported at least 20 seizures of a seizure type)**.** Aware seizure types are shown on the left for P1, P5, P6, P29 and P31. On the right, impaired awareness, motor seizures are shown for P31; aware, motor seizures are shown for P31; and aware, non-motor seizures are shown for P23. Black lines (shading) show average (standard deviation) heart rate at different times of the day across whole dataset, excluding pre- and post-ictal seizure times. Green crosses show average pre-ictal heart rate for each seizure reported of a particular type. Orange crosses show average post-ictal heart rate for each seizure reported of a particular type. Pre- and post-ictal are defined as 1 hour before and after a seizure was reported, respectively. Note that all x axes are the same (time of day).

References

1. Torrence C, Compo GP. A practical guide to wavelet analysis. *Bulletin of the American Meteorological society*. 1998;79(1):61-78.

2. Ajne B. A simple test for uniformity of a circular distribution. *Biometrika*. 1968;55(2):343-354.

3. Abdi H. Bonferroni and Šidák corrections for multiple comparisons. *Encyclopedia of measurement and statistics*. 2007;3:103-107.

4. Leguia MG, Rao VR, Kleen JK, Baud MO. Measuring synchrony in bio-medical timeseries. *Chaos: An Interdisciplinary Journal of Nonlinear Science*. 2021;31(1):013138.

5. Haut SR. Seizure clusters: characteristics and treatment. *Current opinion in neurology*. 2015;28(2):143-150.

6. Karoly PJ, Goldenholz DM, Freestone DR, et al. Circadian and circaseptan rhythms in human epilepsy: a retrospective cohort study. *The Lancet Neurology*. 2018;17(11):977-985. doi:10.1016/S1474-4422(18)30274-6

7. Cook MJ, Varsavsky A, Himes D, et al. The dynamics of the epileptic brain reveal long-memory processes. *Frontiers in neurology*. 2014;5. Accessed October 18, 2017. https://www.ncbi.nlm.nih.gov/pmc/articles/PMC4208412/
